# Spontaneous Formation and Fusion of Raspberry Vesicle Self-Assembled from Star Block Terpolymers in Aqueous Solution

**DOI:** 10.3390/ma14247690

**Published:** 2021-12-13

**Authors:** Yingying Guo, Shuyan Yang

**Affiliations:** 1 School of Science, Qingdao University of Technology, 777 JLJ Road, Qingdao 266520, China; 2 School of Mechanical and Automotive Engineering, Qingdao University of Technology, 777 JLJ Road, Qingdao 266520, China

**Keywords:** raspberry vesicle, vesicle formation mechanism, fusion, dissipative particle dynamic

## Abstract

The spontaneous formation and fusion of raspberry vesicles was studied using the dissipative particle dynamics (DPD) method. The vesicles were formed through the self-assembly of amphiphilic E_12_O_6_F_2_ star terpolymers in selective solvent. E and F blocks are solvophobic and the O block is solvophilic. The shortest F block plays a major role in the formation of raspberry vesicles. Distinct vesicle formation mechanisms were observed at different polymer concentrations. At higher concentrations, vesicles form via the bending and closure of an oblate F-bump-E bilayer. At lower concentrations, the formation pathway contains: the initial formation of a vesicle with a core, the combination of such vesicles into cylindrical micelles, and the bending of the cylindrical micelles to form a hollow vesicle. In addition, raspberry vesicle fusion is regulated by F bumps through the continuous coalescence of them from apposed vesicle membranes. The contact area bends, followed by the formation of a fusion pore and a tilted inner layer. As the pore sealed, the hemifusion structure appears, which further restructures to form a vesicle. Our results provide guidance on understanding the dynamic processes of complex vesicles and biological membrane fusion.

## 1. Introduction

Micelles, whose surfaces are composed of physically or chemically distinct domains, are regarded as soft patchy micelles [1,2]. Their multi-compartment features make them suitable templates for selective nanoparticle incorporation [3]. Additionally, micelles could also be utilized as building blocks for the directional assembly of periodic superstructures [4,5], thus paving the way for application into memory or optoelectronic devices [5]. Distinct from patchy micelles, patchy capsules (vesicles) possess both functionalities of patchy micelles as well as those of vesicles [6]. As a result, such vesicles are potential candidates for medical, biotechnological, or sensor applications.

As a carrier, optimizing vesicle encapsulation efficiency is crucial. The loading efficiency has been found to be affected by the vesicle formation mechanism. The most commonly investigated formation mechanism (Mechanism I) can be described as the wrapping-up of a disk or lamellar micelle, where the solvent and cargo molecules are encapsulated at the same time as the formation of the cavity of the vesicle. In Mechanism II, vesicles form through the diffusion of the solvent into semivesicles, and a cavity is formed as a result. Vesicles were also found to be formed by torus micelles [7]. However, vesicles obtained from Mechanism I are supposed to have higher encapsulation efficiency [8]. Thus, controlling the vesicle formation path is of great importance to improving encapsulation efficiency. Studies [7,9,10,11] have revealed that by manipulating conditions such as solvent addition rate, polymer concentration, block ratio, polymer–solvent interaction, or heating rate, the formation mechanism can be tuned accordingly.

Fusion behavior is another important feature of vesicles [12,13,14]. Vesicle fusion is another way to improve the encapsulation ability by enlarging the vesicle size. Recently, fusion was found to be a method for the formation of large compound vesicles (LCVs) [15]. Much effort through both simulation and experiment [16,17,18,19,20,21,22,23] has been undertaken to understand the fusion process of polymer vesicles with simple unilamellar structure. Several models regarding the fusion process have been reported. In the original stalk-pore mechanism, the inner monolayers of two vesicles merge. The contact area expands further before the formation of a small pore. However, for the modified model, a pore forms directly at the trans-monolayer contact area without expanding the contact area. An atypical path called the stalk-bending process was also observed for vesicles self-assembled from comb-like block copolymers [23]. In this process, two holes appear in both vesicles near the foot of the stalk. The stalk bends and encircles the two holes to complete the fusion process.

The development of polymerization techniques has increased the variety of polymer chain architectures (e.g., linear, star, graft, and dendrimer) as well as the number of building blocks (e.g., ABC triblock), which lead to the formation of polymer vesicles with complex structures, e.g., nanostructured vesicles, patchy vesicles, multilayer vesicles, and Janus dendrimersomes [6,12,13,24,25,26]. The complexity of the vesicle structure could lead to a different fusion path. For example, the fusion process for multilamellar vesicles (MLVs) was found to be layer-by-layer, which starts at the outmost layer and ends at the innermost one. Nevertheless, little attention has been paid to the fusion of vesicles with complex structures. Since many functions of vesicles are related to their fusion behavior [23], investigating the fusion process can help us understand the relationship between structures and the design of potential applications [13,27].

As reported from both experiments and simulations, patchy hollow capsules can be obtained from the self-assembly of amphiphilic block copolymers in selective solvent [25,28,29,30]. In this study, the self-assembly of highly asymmetric E_12_O_6_F_2_ miktoarm star terpolymers in selective solvent was investigated using dissipative particle dynamics simulation. In the miktoarm structure, polymer arms are linked at a single point, which suppresses the formation of concentric structure [31]. The relatively short F block makes the final equilibrium morphologies prone to forming F-block patches. We investigated how the morphologies evolve by systematically modifying the solvophobicity of E and F blocks. The vesicle formation mechanism was studied in different polymer concentrations. The fusion path of the patchy vesicle was also studied for further understanding of the dynamic processes of complex vesicles.

## 2. Simulation Methods

In this simulation, dissipative particle dynamics (DPD) method [32] was conducted to study the spontaneous formation process of the polymer vesicles. The motion of DPD particles is governed by Newton’s equation of motion,

(1) $$\frac{d{\vec{r}}_{i}}{dt}={\vec{\upsilon }}_{i},\;m_{i}\frac{d{\vec{v}}_{i}}{dt}={\vec{f}}_{i}$$

where ${\vec{f}}_{i}$ is the total force exerted on particle i. In DPD simulation [33], the total force consists of four kinds of forces: conservative force (${\vec{F}}_{ij}^{C}$), dissipative force (${\vec{F}}_{ij}^{D}$), random force (${\vec{F}}_{ij}^{R}$), and spring force (${\vec{F}}_{ij}^{S}$). The former three forces are pairwise contributions and become effective when the distance between two beads is within the cut-off radius. The last one is the simple harmonic spring force acted on the bonded beads. The conservative force ${\vec{F}}_{ij}^{C}$ for nonbonded beads is a soft-repulsive force and is given by

(2) $${\vec{F}}_{ij}^{C}=\{\begin{array}{c} a_{ij}\left(1-r_{ij}/r_{c}\right){\hat{r}}_{ij}\;\;\;\;\;\;\;\;\;\;(r_{ij}<r_{c})\;\\ 0\;\;\;\;\;\;\;\;\;\;\;\;\;\;\;\;\;\;\;\;\;\;\;\;\;\;\;\;\;\;\;\;\;\;\;\;\;\;\;\left(r_{ij}\geq r_{c}\right)\; \end{array}$$

where$\;{\vec{r}}_{ij}={\vec{r}}_{i}-{\vec{r}}_{j},r_{ij}=\left|{\vec{r}}_{ij}\right|,{\hat{r}}_{ij}={\vec{r}}_{ij}/\left|{\vec{r}}_{ij}\right|$, $a_{ij}$ is the maximum repulsion between bead i and j, and $r_{c}$ is the cut-off radius with a value of 1.0. The soft-repulsive force used here allows the simulation to have a larger length scale and timescale. The combination of dissipative force and random force plays a role as a thermostat in the simulation. The two forces are given by the following expressions:

(3) $${\vec{F}}_{ij}^{D}=-\gamma \omega^{D}\left(r_{ij}\right)\left({\vec{\upsilon }}_{ij}•{\hat{r}}_{ij}\right){\hat{r}}_{ij}$$

(4) $${\vec{F}}_{ij}^{R}=\sigma \omega^{R}\left(r_{ij}\right)\frac{\xi_{ij}}{\sqrt{dt}}{\hat{r}}_{ij}$$

where ${\vec{\upsilon }}_{ij}={\vec{\upsilon }}_{i}-{\vec{\upsilon }}_{j}$; σ and γ represent the amplitude of the dissipative and random forces, respectively; $\omega^{D}\left(r_{ij}\right)$ and $\omega^{R}\left(r_{ij}\right)$ are the weight functions; $\xi_{ij}$ is a random number with zero mean and unit variance; the $\sqrt{dt}$ is used here to ensure that the diffusion coefficient of the particles is independent of the timestep used in the simulation. In order to satisfy the equilibrium Gibbs–Boltzmann distribution and the fluctuation-dissipation theorem, the following two relations must be satisfied:

(5) $$\omega^{D}\left(r_{ij}\right)={\left[\omega^{R}\left(r_{ij}\right)\right]}^{2},\;\sigma^{2}=2\gamma k_{B}T.$$

According to Groot and Warren [33], γ was chosen to be 4.5 at density ρ=3. The weight function is generally expressed as:

(6) $$\omega^{D}\left(r_{ij}\right)=\{\begin{array}{c} {\left(1-r_{ij}/r_{c}\right)}^{2}\;\;\;\;\;\;\;\;\;\;\;\;\;\;\;\;\;\;\;\;\;\;(r_{ij}<r_{c})\\ 0\;\;\;\;\;\;\;\;\;\;\;\;\;\;\;\;\;\;\;\;\;\;\;\;\;\;\;\;\;\;\;\;\;\;\;\;\;\;\;\;\;\;\;\left(r_{ij}\geq r_{c}\right) \end{array}\;\;\;\;\;\;\;\;\;\;\;\;\;\;\;\;\;\;\;\;\;\;\;\;\;\;\;\;\;$$

The spring force is given by:

(7) $${\vec{F}}_{i}^{S}=\underset{j}{\sum }C{\vec{r}}_{ij}$$

where C is the spring constant and set as 4.0. This value results in a slightly smaller distance for bonded particles than nonbonded ones, and the sum runs over all particles to which particle i is connected.

The integration of the equation of motion (Equation (1)) was carried out using the Velocity–Verlet algorithm [33] with a timestep of Δt=0.04. For simplicity, we set the cutoff radius, the particle mass, and the temperature as the units of the simulation system, i.e., $r_{c}=m=k_{B}T=1.0$. Therefore, the unit of time is$\;\tau =r_{c}\sqrt{m/k_{B}T}=1.0$. Periodic boundary conditions and NVT ensemble were adopted in the simulation. For ρ=3.0, $a_{ij}$ is chosen according to the linear relationship with Flory–Huggins χ parameter proposed by Groot and Warren [33,34].

(8) $$a_{ij}=a_{ii}+3.27\chi_{ij}$$

**Figure 1:**
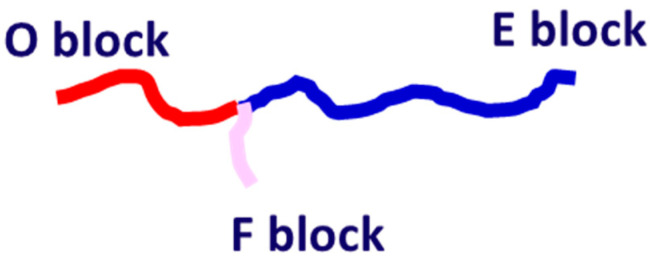
Schematics of the star block copolymers and color codes of individual polymer blocks.

In this study, we built a coarse-grained model for highly asymmetric amphiphilic star E_12_O_6_F_2_ triblock copolymer (Figure 1) with a solvophilic O block. We first studied the effect of the interaction energy between polymer and solvent on the formation of patchy vesicles. In this case, the concentration was set to 10.0 vol %. The interaction energy between the E block with solvent varied from 26.0 to 98.0, and the F block with solvent varied from 26.0 to 126.0, in order to investigate the effect of solvophobicity of the long block and short block on the self-assembly morphologies. We then studied the effect of polymer concentrations on the vesicle formation mechanism. In this case, we used the maximum interaction energy between the solvophobic blocks and solvent ($a_{ES}=97.9;a_{FS}=125.0$). The interaction parameters among the species are summarized in Table 1. We mainly performed the system with a simulation box of $40r_{c}\times 40r_{c}\times 40r_{c}$, and 5.0 × 10^5^ to 8.0 × 10^5^ DPD steps were taken to guarantee the equilibrium state. To study the dynamic process of vesicle fusion, we expanded the simulation box to a size of $70r_{c}\times 70r_{c}\times 70r_{c}$, and 2.0 × 10^7^ DPD steps were taken to guarantee the equilibrium of the fusion process.

## 3. Results and Discussion

### 3.1. Effect of Block Solvophobicity on Vesicle Formation

**Figure 2:**
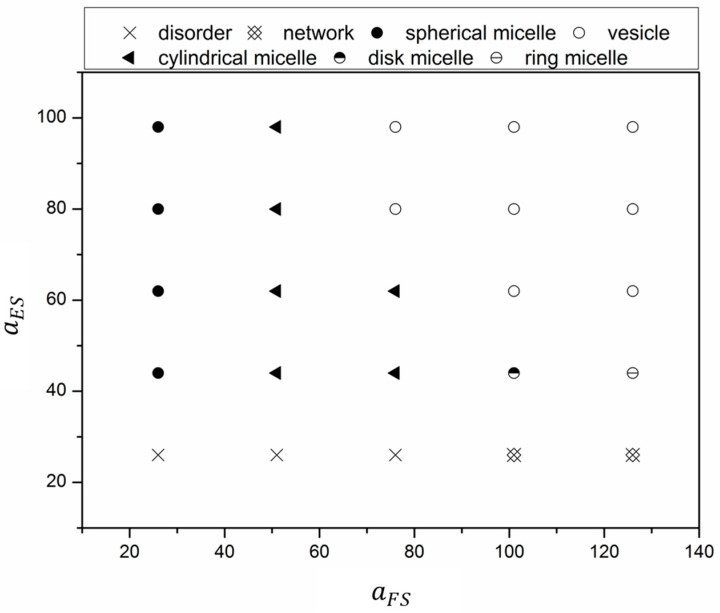
Effect of solvophobicity of longest E block and shortest F block on the equilibrium morphologies.

To illustrate the effect of the interaction energy between blocks and solvents, equilibrium structures for various $a_{FS}$ and $a_{ES}$ are presented in Figure 2 in the form of a state diagram. At low $a_{ES}$ ($a_{ES}\;$= 26), when the interaction energy between the F block and solvent was low ($a_{FS}\leq \;$76), polymer chains favored the solvent, and a disordered structure was found in these cases. As $a_{FS}$ increased from 111.0 to 126.0, the F blocks became relatively solvophobic and started to aggregate. Since E blocks and O blocks are solvophilic, the chains swelled in the solvent and entangled to form a network structure. For cases with higher $a_{ES}$ ($a_{ES}\;$> 26), the equilibrium morphologies were mainly governed by short F blocks when $a_{FS}$ was low ($a_{FS}\leq 51$). Polymer chains self-assembled to spherical micelles at low $a_{FS}$ ($a_{FS}\;$= 26), while cylindrical micelles did so at high $a_{FS}$ ($a_{FS}\;$= 51). For further increased $a_{FS}$, low and high $a_{ES}$ favored the formation of micelles and vesicles, respectively. Although amphiphilic polymers could self-assemble into vesicles with hydrophilic fraction 35% ± 10%, our results showed that the solvophobicity of the hydrophobic blocks also played an important role in vesicle formation. Note that the length ratio between E blocks and F blocks was 6:1; thus, the solvophobicity of the shortest F blocks played a more important role in the formation of vesicles. 

### 3.2. Vesicle Formation Mechanism

**Figure 3:**
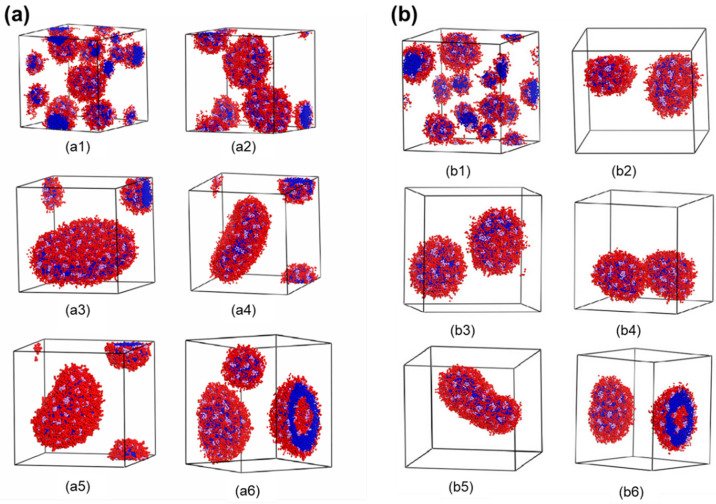
(**a**) Typical formation process of raspberry vesicle self-assembled from E_12_O_6_F_2_ with a polymer concentration of $f_{p}=0.15$ at (**a1**) 50,000 DPD steps; (**a2**) 150,000 DPD steps; (**a3**) 175,000 DPD steps; (**a4**) 200,000 DPD steps; (**a5**) 210,000 DPD steps; and (**a6**) 250,000 to 500,000 DPD steps. (**b**) Typical formation process of raspberry vesicle self-assembled from E_12_O_6_F_2_ with polymer concentration of $f_{p}=0.1$ at (**b1**) 50,000 DPD steps; (**b2**) 280,000 DPD steps; (**b3**) 350,000 DPD steps; (**b4**) 424,000 DPD steps; (**b5**) 470,000 DPD steps; and (**b6**) 500,000 to 800,000 DPD steps. The blue, red, and pink colors represent E, O, and F, respectively, while solvent molecules are omitted for clarity.

**Figure 4:**
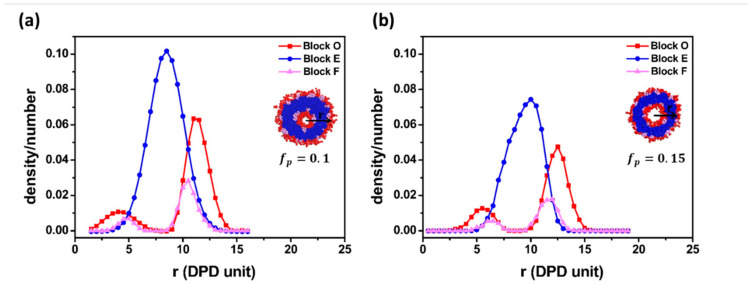
Composition profiles of vesicles formed in different polymer concentrations: (**a**) *f_p_* = 0.1; (**b**) *f_p_* = 0.15.

Polymer concentration plays an important role in the vesicle formation path. In this paper, polymer concentration is represented as $f_{p}$. Figure 3a,b illustrates the typical vesicle formation processes for E_12_O_6_F_2_ star terpolymers at two different polymer concentrations. In the case of higher terpolymer concentration ($f_{p}=0.15$), amphiphilic block copolymers first aggregated into spherical raspberry micelles (Figure 3(a1,a2)). The spherical micelles merged and restructured to form an oblate F-bump-E bilayer. The bilayer further curled up into a complete vesicle (Figure 3(a3–a6)). In this case, the vesicle was formed through a lamellar micelle closure process, a common path reported in the literature as Mechanism I [7,10]. Figure 3b displays the dynamic process of vesicle formation at a lower terpolymer concentration ($f_{p}=0.1$). As referred to in Figure 3(b1), the aggregation of star terpolymers went through a similar initial stage as shown in Figure 3(a1). Disk-like micelles appeared due to the combination of the small spherical micelles (Figure 3(b2)). As time increased, the disk-like micelle gradually transformed into a vesicle with a core (Figure 3(b3)). Those aggregates were stable for a period. After 74,000 DPD steps, the vesicle with a core collided and the morphology finally evolved into a hollow vesicle (Figure 3(b4–b6)). The final structures (cross-section) and density profiles of vesicles obtained from both high and low concentrations are shown in Figure 4. Both density profiles apparently showed bimodal characteristics for the hydrophilic O block and the hydrophobic F block, while unimodal features were found in the hydrophobic E block. This suggested that the membrane was made of E block. The solvophilic O block was located both inside and outside the membrane, which suggested the typical nature of a vesicle. The super solvophobic F blocks aggregated into small clusters and bumped to both sides of the membrane. The raspberry vesicle structure was formed due to the relatively large chain ratio between blocks E and F. Thus, E blocks exhibited more solvophobic behavior than the solvophobic F blocks. In conclusion, the two final vesicle structures were similar, with exceptions in the distinct formation pathways adopted at different polymer concentrations. Such a difference was realized as a result of the increase in collision probability between adhesive micelles that resulted from the increase in polymer concentration [10].

**Figure 5:**
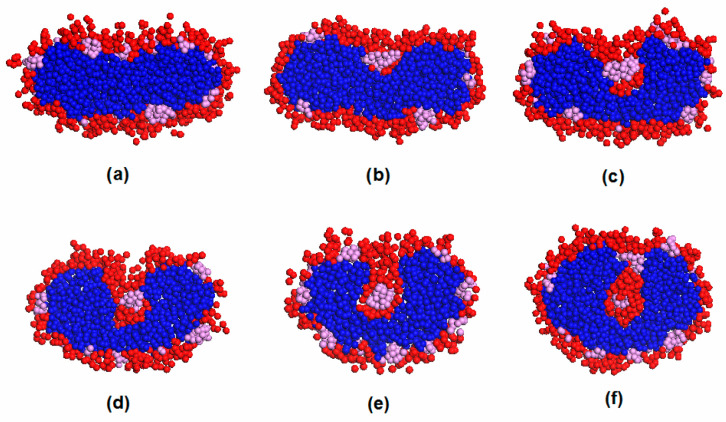
Snapshots for the morphology transition from disk-like micelle to vesicle with a core over the period of 2.8 × 10^5^ DPD steps to 3.5 × 10^5^ DPD steps. (**a**) 280,000 DPD steps; (**b**) at 290,000 DPD steps; (**c**) at 300,000 DPD steps; (**d**) at 320,000 DPD steps; (**e**) at 340,000 DPD steps; and (**f**) at 350,000 DPD steps. The blue, red, and pink colors represent E, O, and F, respectively, while solvent molecules are omitted for clarity.

**Figure 6:**
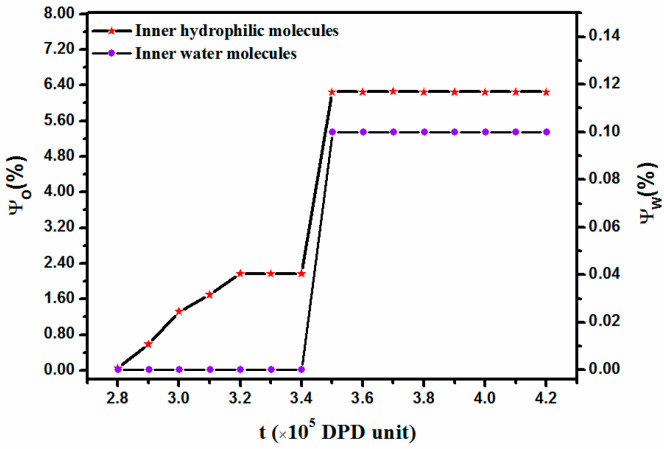
Diffusion number of solvophilic block O and solvent varies with time. $\phi_{O}$: number of interior solvophilic O-beads as percent of all solvophilic molecules of the structure (**left**); $\phi_{W}$: number of interior solvents as percent of all molecules of the structure (**right**).

For clarity, the morphology transition from a disk-like micelle to a vesicle with a core over the period of 2.8 × 10^5^ to 3.5 × 10^5^ DPD steps at a low concentration is displayed in Figure 5, in which an interesting finding is observed regarding its structural evolution. For more simplicity, only the cleaved structure of the aggregate is exhibited. As shown in Figure 5a, the micelle exhibited a disk-like structure with an E-bead center surrounded by a shell made mainly of O and F blocks. This stage was followed by the diffusion of solvophilic O blocks into the micelle interior. To better understand the evolution process, the variations in the diffusion number of the solvophilic O block and solvent as a function of time are displayed in Figure 6. In the figure, $\phi_{O}$ represents the number of interior solvophilic O beads as a percentage of all solvophilic molecules of the micelle and $\phi_{W}$ represents the number of interior solvent content as a percentage of all micelle molecules. Figure 6 clearly shows that the number of interior solvophilic O beads steadily increased over the period of 2.8 × 10^5^ to 3.2 × 10^5^ DPD steps, which corresponds to the dynamic processes described in Figure 5a–d. This process is similar to the previously described Mechanism II, in which the solvophilic block in the exterior of the micelle diffuses into the center (nucleation) and results in the formation of a semivesicle. Since the O beads gradually diffused toward the center of the aggregate, the central E beads were excluded to the sides, which led to the bending of the semivesicle (Figure 5d). Once the semivesicle reached a certain curvature, the diffusion of O beads stopped, shown in Figure 6, in which a constant value (about 2.1%) for the number of O beads inside the structure (from 3.2 × 10^5^ to 3.4 × 10^5^ DPD steps) was observed. The curved semivesicle closed spontaneously. The closure of the structure resulted in a step change for the percentage of inner O-beads in the micelle (Figure 6). According to Figure 6, solvent molecules, though not many, were found inside the structure after the closure of the semivesicle. Learning from Figure 5f and Figure 6, we know that the vesicle with a hydrophobic F block core formed during this stage. Such a special structure was also reported by Wang et al. in a study of the self-assembly of star terpolymers by the SCFT method [29]. In their work, a vesicle with a core was formed due to the aggregation of shorter solvophobic blocks at the center during vesicle size enlargement. In our simulation, the hydrophobic core was formed due to the intrinsic polymer structure. When the O block diffused into the micelle center, the F block, which connected with the O block, deposited simultaneously (Figure 5b–d). The structure was stable for about 74,000 DPD steps. 

**Figure 7:**
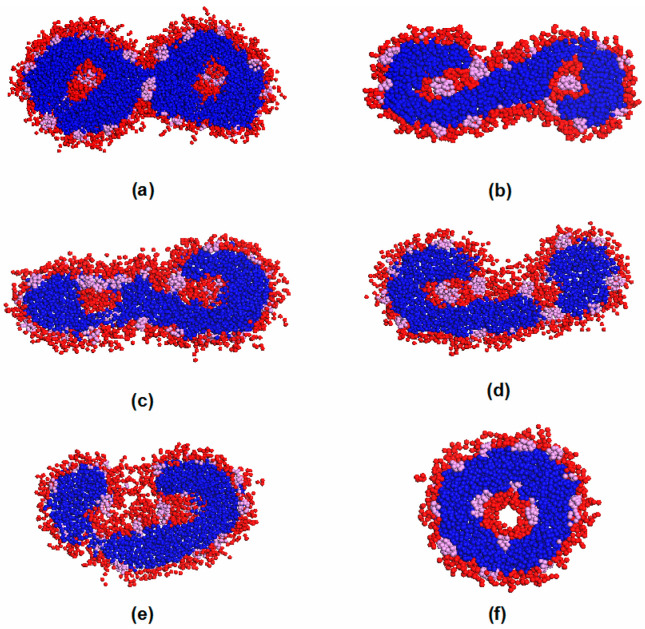
Morphology transition during the combination of two vesicles with a core over the periods of 4.24 × 10^5^ DPD steps to 5.0 × 10^5^ DPD steps: (**a**) at 424,000 DPD steps; (**b**) at 430,000 DPD steps; (**c**) at 450,000 DPD steps; (**d**) at 470,000 DPD steps; (**e**) at 485,000 DPD steps; and (**f**) at 500,000 DPD steps. The blue, red, and pink colors represent E, O, and F, respectively, while solvent molecules are omitted for clarity.

**Figure 8:**
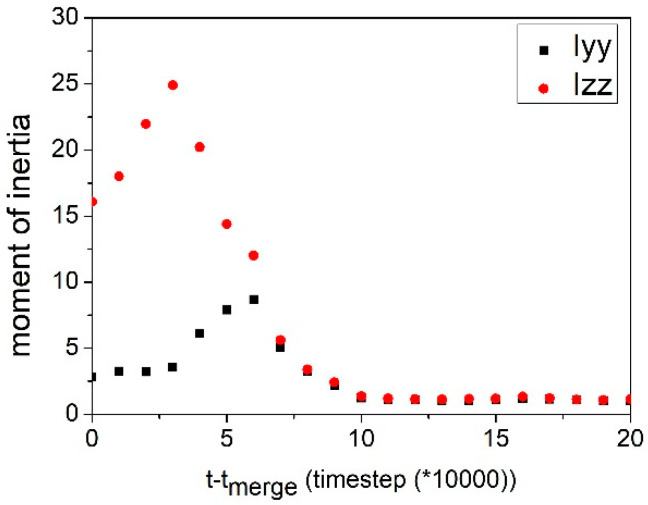
Shape characterization by moment of inertia after merging. $I_{xx}$, $I_{yy}$, and $I_{zz}$ are the principal moments of inertia of the aggregate.

Figure 3(b5) shows the formation of a cylindrical micelle. The micelle was formed by the collision of two vesicles. Both vesicles had a hydrophobic core inside. Figure 7 shows the cross-sections of morphology transition after the combination of the two vesicles. The compatibility among the three blocks thereby led to a gradual rearrangement of polymer composition and curvature (Figure 7b–d). As a result, a complete hollow vesicle formed (Figure 7f). The morphology transition from a cylindrical micelle to a vesicle was also observed by Narayanan et al. [7] at a lower polymer concentration. Nevertheless, the evolution process from a cylindrical micelle to a hollow vesicle in our study was different. For clarity, the shape transformation of the micelles is evaluated by its principal moments of inertia, $I_{xx},\;I_{yy},\;\mathrm{and}\;I_{zz}$; here, we use the normalized principal moments of inertia, $\frac{I_{yy}}{I_{xx}}$ and $\frac{I_{zz}}{I_{xx}}$. Typically, spherical aggregates would have $\frac{I_{yy}}{I_{xx}}$ and $\frac{I_{zz}}{I_{xx}}\approx$ 1, while $\frac{I_{yy}}{I_{xx}}<\frac{I_{zz}}{I_{xx}}$ and $\frac{I_{yy}}{I_{xx}}\approx \frac{I_{zz}}{I_{xx}}>1$ correspond to a cylinder and a disk, respectively. In Figure 8, two parent micelles touch at $t-t_{merge}$ = 0 (corresponding to Figure 3(b4)), forming a daughter micelle, a structure with long $I_{zz}$, short $I_{yy}$, and $I_{xx}$. The resulting aggregate elongated in length to form a cylindrical micelle. The cylindrical micelle restructured and curved to form a spherical vesicle. In the reported experiment, the cylindrical micelle first grew to a certain length and formed a torus-like micelle. Then, the torus micelle merged to form a hollow structure. In our simulation, we did not observe the formation of a torus micelle. Overall, the diffusion of solvophilic O blocks caused the structural transition from a disk-like micelle to a vesicle with a core. Such vesicles merged to form a cylindrical structure. The cylindrical structure eventually bent to form a hollow vesicle. The vesicle with a core could be regarded as an intermediate state during our vesicle formation process.

### 3.3. Vesicle Fusion

**Figure 9:**
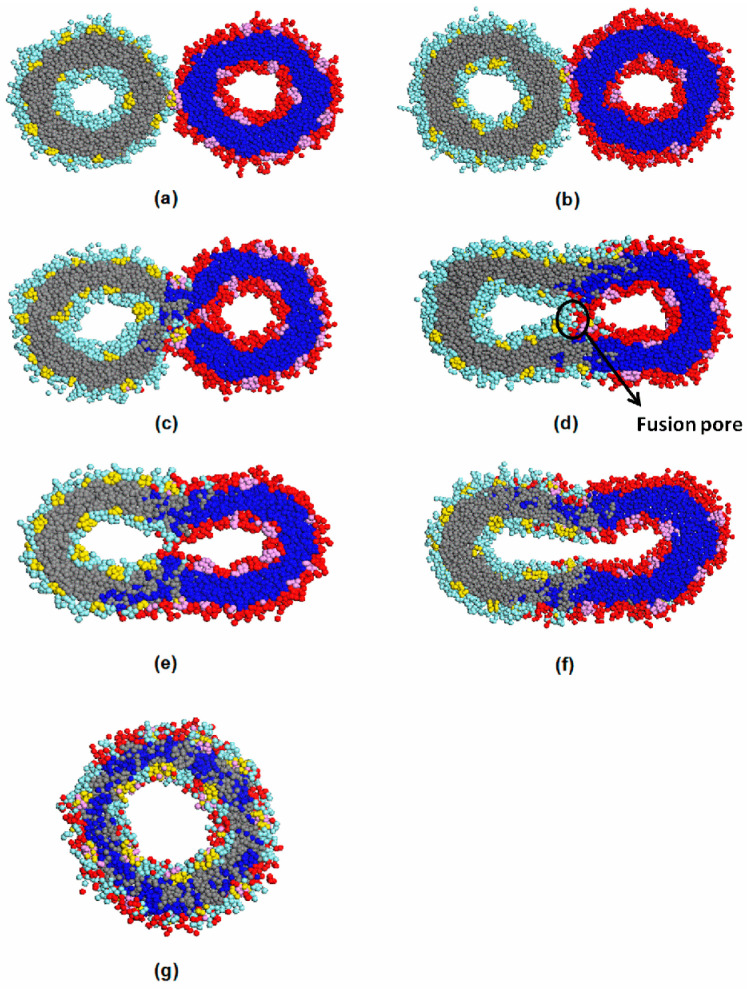
Dynamic of vesicle fusion at (**a**) 250,000 DPD steps; (**b**) 350,000 DPD steps; (**c**) 555,000 DPD steps; (**d**) 660,000 DPD steps; (**e**) 850,000 DPD steps; (**f**) 1,000,000 DPD steps; and (**g**) 20,000,000 DPD steps. Colors of grey and blue, light blue and red, and yellow and pink represent block E, block O, and block F for two different vesicles, respectively. Solvent molecules are omitted for clarity.

In order to study vesicle fusion, two vesicles were put in one large simulation box. For clarity, different colors are adopted for the two vesicles, depicted in Figure 9. The fusion process initiated with the merging of F bumps from opposite vesicle membranes (Figure 9a). As reported [35], there are two fusion mechanisms for lipid membrane fusion. One suggests that the fusion is modulated by membrane proteins through forming continuous proteinaceous pores between apposed membranes. The F bumps in our raspberry vesicle could be viewed as proteins embedded in the membrane. As observed from Figure 9a,b, opposite F bumps coalesced between apposed vesicle membranes. Thus, our fusion process was regulated by F bumps and our simulation supported the aforementioned lipid fusion mechanism. The contact area between the two vesicles then further expanded. Some of the F bumps and solvophilic O blocks formed a small cluster. The cluster was embedded into one of the vesicle membranes (Figure 9c). We ascribed this to the bending of the contact area during the membrane combination. The bending may have been promoted by the connection of F bumps from each vesicle, which led to an uneven distribution of the membrane curvature. Contact area bending has also been reported by Brownian dynamic simulation [36] and molecular dynamics simulation [37] of lipid vesicles. The cluster gradually mixed with the inner layer and finally disappeared. A pore was then generated at the center of the contact area (Figure 9d). The current structure could then be regarded as a stalk. In this stage, in order to solve the high energy of the stalk intermediate [38], the solvophobic chains tilted, and a sharp corner could be found at the distal of both inner layers. As time increased, hemifusion intermediates formed after the pore seals (Figure 9e). The intermediate further expanded into a tubular vesicle (Figure 9f). Finally, the tubular vesicle restructured into a larger spherical raspberry vesicle (Figure 9g). Our results in general agree with the second mechanism obtained from the molecular dynamic simulation of lipid vesicles [38]. The number of solvent molecules before and after fusion was also calculated to understand the dynamic process. In contrast to the reported vesicle leakage after fusion finished [19,38], there was a 0.24% increase in solvent molecules after complete fusion in our simulation. The reason may be attributable to the bending of the contact area, which encapsulated some solvent molecules simultaneously during the fusion process. 

## 4. Conclusions

In this paper, we applied the dissipative particle dynamics (DPD) method to investigate the formation and fusion mechanism of raspberry vesicles that self-assembled from amphiphilic EOF star terpolymers in selective solvent. The work showed that the solvophobicity of short F blocks plays a major role in vesicle formation. As well, distinct formation paths were observed in different polymer concentrations. Vesicles form through the wrapping-up of lamellar micelles in higher polymer concentration. However, in lower concentrations, the vesicles form through the restructuring of a cylindrical micelle. The cylindrical micelle is obtained from the collision of vesicles with a hydrophobic core. The elongation and uneven distribution of curvature play a critical role in the morphology transition from a cylindrical micelle to the hollow structure. For raspberry vesicle fusion, the process includes: (1) kissing of F bumps from apposed membranes; (2) formation and bending of a contact area; (3) formation of a pore in the center of the contact area; (5) expansion of the pore to form a hemifusion structure; (6) formation of a tubular vesicle and restructuring of the vesicle. Additional solvent molecules are encapsulated after fusion completed. Since the F bumps in the raspberry vesicle can be viewed as proteins embedded in the lipid membrane, our results provide new guidance on understanding the dynamic formation and fusion of the biological membrane.

## Figures and Tables

**Table 1:** Interaction parameters $a_{ij}$ (in DPD units) used in the simulations.

Type of Blocks	O	E	F	S
O	25.0			
E	38.5	25.0		
F	89.4	78.0	25.0	
S	26.0	26.0–97.9	26.0–125.0	25.0

## Data Availability

Not applicable.
